# A Genetic Algorithm for the Generation of Packetization Masks for Robust Image Communication

**DOI:** 10.3390/s17050981

**Published:** 2017-04-28

**Authors:** Katherine Zapata-Quiñones, Cristian Duran-Faundez, Gilberto Gutiérrez, Vincent Lecuire, Christopher Arredondo-Flores, Hugo Jara-Lipán

**Affiliations:** 1 Magister en Ciencias de la Computación, Universidad del Bío-Bío, Chillán 3800708, Chile; kzapata@ubiobio.cl; 2 Departamento de Ingeniería Eléctrica y Electrónica, Universidad del Bío-Bío, Concepción 4051381, Chile; 3 Departamento de Ciencias de la Computación y Tecnologías de la Información, Universidad del Bío-Bío, Chillán 3800708, Chile; ggutierr@ubiobio.cl; 4 Centre de Recherche en Automatique de Nancy, Université de Lorraine, CNRS, Vandœuvre-lès-Nancy 54506, France; vincent.lecuire@univ-lorraine.fr; 5 Magister en Informática, Universidad del Bío-Bío, Concepción 4051381, Chile; carredon@alumnos.ubiobio.cl; 6 Corporación Educacional Colegio Concepción, Nuble 3800564, Chile; hjara@cocochi.cl

**Keywords:** robust image communication, packetization method, block interleaving, genetic algorithms, camera sensor networks

## Abstract

Image interleaving has proven to be an effective solution to provide the robustness of image communication systems when resource limitations make reliable protocols unsuitable (e.g., in wireless camera sensor networks); however, the search for optimal interleaving patterns is scarcely tackled in the literature. In 2008, Rombaut et al. presented an interesting approach introducing a packetization mask generator based in Simulated Annealing (SA), including a cost function, which allows assessing the suitability of a packetization pattern, avoiding extensive simulations. In this work, we present a complementary study about the non-trivial problem of generating optimal packetization patterns. We propose a genetic algorithm, as an alternative to the cited work, adopting the mentioned cost function, then comparing it to the SA approach and a torus automorphism interleaver. In addition, we engage the validation of the cost function and provide results attempting to conclude about its implication in the quality of reconstructed images. Several scenarios based on visual sensor networks applications were tested in a computer application. Results in terms of the selected cost function and image quality metric PSNR show that our algorithm presents similar results to the other approaches. Finally, we discuss the obtained results and comment about open research challenges.

## 1. Introduction

It is known that, when sending multimedia data through communication networks, the inherent presence of packet loss can lead to discernible quality degradations at the receiver side [1]. Moreover, when the Packet Loss Rate (PLR) is significant, loss control becomes a relevant issue for reliable (or semi-reliable) communication protocols, being commonly addressed with active concealment techniques [2], such as those based in Forward Error Correction (FEC) or Automatic Repeat-reQuest (ARQ) [3,4]. Nevertheless, the use of such techniques is unsuitable in many applications for which energy, time and/or bandwidth (among others) are considered as very scarce resources. For those cases, even if unable to perform error correction, interleaving methods have proven to be effective on the reduction of the impact of network errors [5].

Interleaving methods take advantage of the usual spacial redundancy of multimedia data. Indeed, neighboring blocks of data are usually similar or highly correlated in multimedia, e.g., in digital images, we expect to find similar intensities in the pixels of a given region representing a single object or area (similar examples be found in sound). Interleaving methods can be used to enhance error robustness by simply spreading neighboring blocks of data in different and distant data packets, so that, if packets are lost, it may be still possible to find the neighboring blocks of those that were lost in other (well received) packets, so the missing information can be better estimated by using a passive error concealment method (e.g., works in [6,7]). This kind of technique has been proposed for being part of energy-aware wireless sensor systems, in real-time video transmission and radio broadcasting, among others [8,9,10,11,12,13].

**Figure 1:**
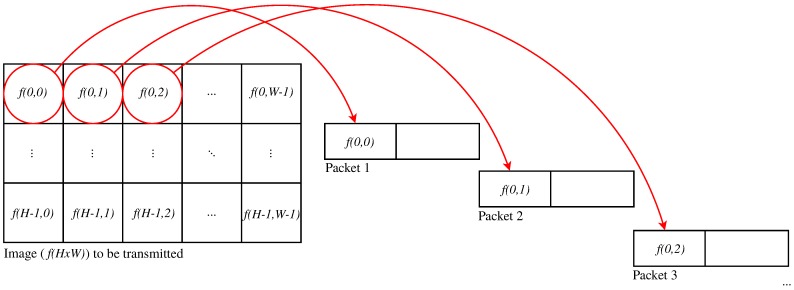
Basic packetization scheme using block interleaving.

This paper focuses on the particular case of two-dimensional still image interleaving, but modifications to the presented proposals can be performed in order to apply them to other kinds of multimedia data (such as sound or video). Let us denote *P* as the number of packets necessary to transmit an image f(H×W). For the rest of this paper, we consider that the packetization scheme searches for sending an integer quantity of the entire data blocks in a packet, so that each of the P−1 first packets is as full as possible, and a data block is either entirely received or entirely lost. Figure 1 shows an example of image interleaving for the said error resilience principle.

Following the example, if Packet 2 is lost, we could find useful information to give an acceptable estimation of f(0,1) original values by using information in Packets 1 and 3 (in the case that these packets are correctly received), assuming that the values of blocks f(0,0) and f(0,2) are fairly similar to those in f(0,1). Now, considering the huge amount of possible solutions, the determination of an optimal packetization scheme through exhaustive search becomes extremely unsuitable. This amount of possible solutions depends on various factors, including the amount and size of the blocks of pixels (codified or not) to be packetized and the size of the packets payload.

In the literature, it is possible to find different approaches for performing interleaving-based packetization, where most of them are based in deterministic mathematical or procedural functions that map an input coordinate (x,y) into another one $\left(x^{\prime },y^{\prime }\right)$. One example is the work in [8], were a Torus Automorphism (TA) was applied as a part of a communication system for resource-constrained wireless camera sensor networks devices. Of course, in the matter of this paper, the effectiveness of the interleaving scheme is assessed by its capability to enhance robustness, hence by its capability to allow a perceptual enhancement of the decoded images at the receiver side. As the number of possible scenarios is impossible to handle by state-of-the-art computational equipment, the finding of a good interleaving function, and the search for its optimal parameters, is a non-trivial task. In the absence of a fitness function able to assess an interleaving scheme, simulations are required. In [11], we performed an experimental comparison of various block interleaving techniques found in the literature, showing results of exhaustive simulations with every possible parameter for each evaluated method, for a large amount of loss patterns over a single input image. Results in terms of Peak Signal-to-Noise Ratio (PSNR) show a slight advantage of TA in front of other interleaving schemes, for the considered scenarios. To the best of our knowledge, this work is the only work in the literature in trying to exhaustively evaluate image interleaving schemes as error-robust techniques; most of the published works provide experimental results with very few cases. Nevertheless, as the required amount of computing time is significant, this experience highlights the need for higher performance computing techniques and, moreover, new evaluation methods.

Avoiding exhaustive simulations for assessing said processing techniques calls for evaluation functions, whose design is not trivial because various considerations must be made. Moreover, the validation of such an evaluation functions is also non-trivial. In [14], the authors present what seems to be the only evaluation function for an interleaving scheme. Such an evaluation function is used as the cost function of a Simulated Annealing (SA) implementation able to find optimal packetization masks.

In this paper, we propose a Genetic Algorithm (GA) for the generation of pseudo-optimal packetization masks, as an alternative to the method proposed by Rombaut et al. in [14], for enhancing error resilience in image transmission schemes. In particular, the contributions of this work are twofold: Firstly, we design the GA capable of calculating packetization masks adopting Rombaut’s evaluation function, and we compare it with Rombaut’s SA and TA, which was reported as a good mathematical interleaving technique [11]. Secondly, we provide exhaustive simulation results for analyzing the relationship between the adopted cost function and the quality of reconstructed images after applying the calculated packetization masks as a part of a virtual communication system subject to packet loss.

The rest of this paper is organized as follows: In Section 2, we present the theoretical background and related works on image interleaving for enhancing error resilience in image communication systems. Section 3 describes the genetic algorithm we designed and implemented in order to generate packetization masks for image communication. Results in terms of the cost function proposed by Rombaut et al. and image quality metric PSNR are discussed in Section 4. Finally, Section 5 concludes and gives future directions of this work.

## 2. Background on Image Interleaving for Error Robust Communication

Due to its simplicity and effectiveness, various works have adopted image interleaving for enhancing image communication robustness. In [15], the coordinate of the following blocks to be packetized is calculated by considering a separation of step blocks in a line-by-line lecture of the captured image. In [16], the authors propose the calculation of the new coordinate for a block to be packetized by applying a Backermap. A similar approach is adopted in [8,17], where a chaotic torus automorphism is applied to enhance the robustness of a real-world wireless camera sensor network.

Given selectable parameters, all of the mentioned approaches represent mathematical functions that map a given initial coordinate to a new one as an injection, i.e, for every input coordinate (x,y), there is only one calculated output coordinate $\left(x^{\prime },y^{\prime }\right)$, and for each input $\left(x^{\prime },y^{\prime }\right)$, we can find the exact original coordinate (x,y) by applying the inverse method (of course, we need to find the original order at the decoder side). As said, calculated coordinate $\left(x^{\prime },y^{\prime }\right)$ is performed to select the next block to be packetized. Even if such techniques are efficient and effective for enhancing robustness in a communication system, the search for optimal packetization patterns is still poorly studied. Indeed, most approaches behave always the same way, but there are no evaluation methods that give us a clue about, given a certain scenario, which interleaving pattern is the most suited.

In [11], we performed exhaustive simulation (i.e., brute-force search, simulating every possible combination of parameter in a certain range) of interleaving methods in [8,16,18], representing hundreds of computing simulation hours. The results of this work throw slight favor to the TA approach based on [8], but the complexity of the evaluation process calls for evaluation functions that allow one to anticipate the suitability of a given approach avoiding such a computing effort. In that matter, an interesting approach is proposed in [14], where an optimization function is proposed in order to assess packetization masks (a particular representation of a packetization pattern) in terms of the robustness that they can provide to an image communication system. This evaluation function is included in an SA algorithm providing pseudo-optimal packetization masks. The evaluation function could also be useful to evaluate block-sampling techniques, such as the work in [19]. In this work, we adopt such a technique and propose a GA as an alternative of the implemented SA. The proposal is compared with a sequential communication and algorithms based on TA [8] and SA [14], which are briefly described in the reminder of this section.

### 2.1. Interleaving Method Based on Torus Automorphisms

Torus Automorphisms (TA) are strongly chaotic systems that can be adapted as a two-dimensional permutation transform [20]. A classical implementation is the one that uses Arnold’s cat map and has been adopted as interleaving function for image communication systems in various published works [8,17,21]. As said, a main quality of such a method is its chaotic results, ensuring pseudorandom interleaving (still being possible to get the original configuration), apart from not incurring excessive energetic waste due to the simplicity of its execution. Equation (1) illustrates the permutation function that calculates a new coordinate $\left(x^{\prime },y^{\prime }\right)$ as function of a previous one (x,y), considering a N×N blocks squared matrix.

(1) x′y′=11kk+1nxymodN

### 2.2. Rombaut Interleaving

In [14], Rombaut et al. present a packetization method introducing a cost function for packetization mask assessing. A packetization mask is a packetization scheme representation consisting of a matrix of integer values f(H×W), where each element $f_{s,t}$ stores the sequence number of the packet transporting the block in coordinates (s,t) from the original image (the data can be codified or not). Rombaut et al.’s proposal is the optimal packetization mask search based on Simulated Annealing (SA) (see Algorithm 1) along with a Metropolis algorithm (see Algorithm 2).

**Algorithm 1** Rombaut et al.’s Simulated Annealing [14].1:Initialize $T_{k}$ with the initial temperature $T_{0}$ {this is a user selectable parameter}.2:Generate an initial packetization mask mask {randomly}.3:**while** the equilibrium is not reached **do**4: [mask,best_mask]← Metropolis($mask,best\_mask,T_{k}$)5: Decrease temperature $T_{k}$. {This can be done by doing $T_{k}\leftarrow T_{0}*\alpha^{k}$, where *k* increases at each iteration.}6:**end while**7:**return** best_mask

**Algorithm 2** Rombaut et al.’s Metropolis algorithm [14].**Require:** Input mask {The current packetization mask (from a previous iteration) which is used as basis for generating a new one.}, input best_mask {Current best packetization mask.}, input $T_{k}$ {Current temperature.} 1:Initialize mask. 2:**while** the equilibrium is not reached **do** 3: Generate a new mask, new_mask, using as basis previous mask mask. 4: Calculate delta_penalty as delta_penalty←penalty(new_mask)−penalty(mask) {penalty() corresponds to to SA’s cost function which must be minimized, and is described in Section 3.3.} 5: Calculate threshold as: $threshold\leftarrow e^{\left(\frac{delta\_penalty}{Tk}\right)}$ 6: **if** rand(1) <threshold {rand(1) calculates a random real number in range [0,1].} **then** 7:  mask←new_mask 8:  **if** threshold>1 **then** 9:   best_mask←new_mask10:  **end if**11: **end if**12:**end while**13:**return** [mask, best_mask]

## 3. Proposal

Genetic algorithms are well-known metaheuristics inspired by Darwin’s theory of evolution. They apply direct analogies of the principle of survival of better adapted individuals to certain environments over time [22]. A GA consists of making a population of individuals, P(t), evolve by making it pass through random processes, as those observed in biological evolution (particularly, crossover and mutation), and a selection process according to a criterion, which allows deciding which individuals are the best adapted [23]. Briefly speaking, a GA randomly generates an initial population of individuals, assigning a fitness value to each one (a calculated value representing its “quality”with respect to the others); then, it applies stochastic operators, such as selection, crossover and mutation, over the population, aiming to generate a new population of individuals. These processes are successively repeated until a certain stopping criterion is accomplished. This stopping criterion can be the execution of a certain number of iterations (generations) or the finding of an individual with a fitness with some desired characteristics.

When developing genetic algorithms, the main components to design are: (1) the codification scheme; (2) the initial population; (3) the fitness function; (4) the selection process; (5) the crossover; (6) the mutation; (7) the replacement (the pass to the next generation); and (8) the stopping criterion. The following subsections describe the components of the proposed GA in order to find suitable solutions to the explained interleaving problem. The adopted codification scheme and the crossover and mutation operators correspond to components adopted in works where it is necessary to maintain all of the elements of a sequence (permutations) consistent [24,25].

### 3.1. Codification Scheme

**Figure 2:**
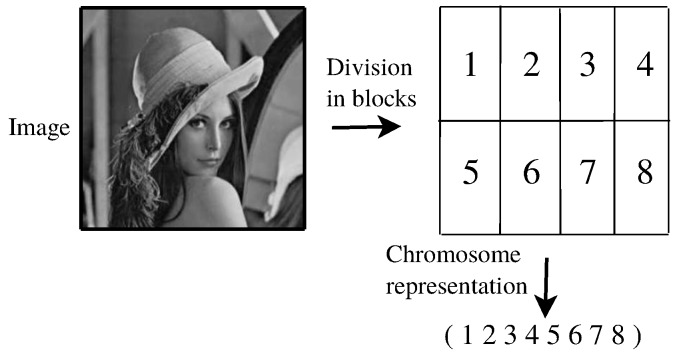
Illustration of the adopted codification scheme to represent a chromosome.

As the codification scheme, we adopt a stream of different integer numbers to represent a given chromosome. The integer numbers symbolize the image’s blocks to be packetized in a line-by-line sequence, such as illustrated in Figure 2. In [25], this kind of codification scheme is considered as a natural representation in problems requiring symbolizing a sequence. In this paper, we represent an individual *I* as a vector of (N×M) different integer numbers, where *N* and *M* are the number of rows and columns of the matrix of blocks to packetize.

Note that each block contains a certain number of pixels. In image processing, many compression algorithms divide the image into blocks of pixels, and then, they apply some transform over each block. For example, the JPEG algorithm works over blocks of 8×8 pixels; the TiBsalgorithm works over blocks of 2×2 pixels [17], and so on. For simplicity (and this is the principle adopted in this paper), many interleaving-based schemes consider packetizing each block as a whole in one packet, so the entire block is either received or lost. With this, as explained before, the quality of a packetization scheme is based on its capacity to increase the probability of receiving neighboring blocks to a lost one considering that neighboring blocks can be used to estimate the intensities of a missing one.

### 3.2. Initial Population

**Figure 3:**
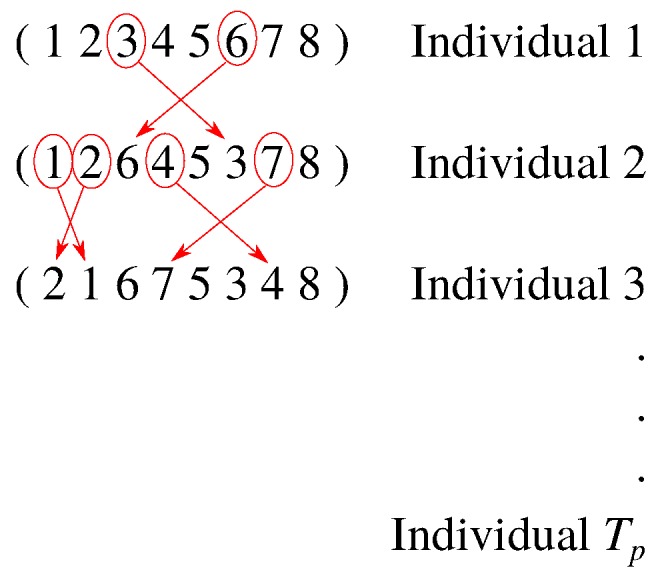
Example of initial population generation.

An initial population of $T_{p}$ candidate solutions is randomly generated from the search space, through random permutations of a vector of (N×M) integer values as illustrated in Figure 3.

### 3.3. Fitness Function

In order to evaluate candidate solutions, in this work, we adopt the cost function proposed by Rombaut et al. in [14], addressed as Rombaut’s Cost Function (RCF). This function evaluates the interpolation mask used for packetization, which is based on properties that, according to the authors, enhance the packetization robustness. RCF is a function to minimize; hence, the closest to zero possible value is the optimal solution.

According to Rombaut et al.’s notation, an image is defined as a signal *f* defined in the discrete domain *D*. According to that, a packetization interpolation mask is defined as a matrix $f=\{f_{0,0},f_{0,1},\ldots f_{N-1,M-1}\}$ where $f_{s,t}$ contains the number of sequences of the data packet in which the block at coordinate (s,t) is transported.

The evaluation function *Q* is thus given by the combination of three terms as described by Equation (2):

(2) $$Q\left(B\right)=\alpha Q_{1}\left(B\right)+\beta Q_{2}\left(B\right)+\gamma Q_{3}\left(B\right)$$

where:

B:    represents the solution,
$Q_{i}\left(\right)$:  (i∈{1,2,3}) corresponds to the properties of a proper packetization, according to Rombaut et al., namely:
  1. a large enough distance between packets, considered by factor $Q_{1}$,
  2. maximal spreading over the packets of the set of elements necessary to reconstruct an element *j*, considered by factor $Q_{2}$, and
  3. maximal spreading of the elements necessary to reconstruct all of the elements of one packet over all of the other packets, considered by factor $Q_{3}$. Finally,
α, β **and** γ:  allow considering the importance of each desired property and are calculated with the knowledge of a determined packet loss model.

Equations for calculating factors $Q_{i}$, α, β and γ are given in the Appendix at the end of this paper.

### 3.4. Selection

After evaluation, the best adapted individuals are selected for the next generation, i.e., solutions with higher fitness values are selected with higher probability than others with lower fitness, so the survival of the best adapted individuals principle is imposed. In our work, we adopt the roulette wheel selection, which consists of assigning to each individual *x* of the current generation a section of size P(x) representing its probability to be chosen, so that ∑P(x)=1, ∀x [26].

### 3.5. Crossover

Crossover combines parts from two or more solutions, in order to create new (and possible better) solutions. Crossover is the operator that gives to genetic algorithms its strength; it allows that different solutions share genetic information; thus, it is expected that, over the generations, the combination of two or more good individuals generates better solutions.

In this work, we adopt Order Crossover (OX1) [27], which is applied to find solutions to the classical Traveling Salesman Problem (TSP) in [24,25], providing good results in comparison with other crossover operators.

OX1 includes an associated crossover probability, $P_{c}$, which determines whether a pair of individuals cross or not. If two individuals, P1 and P2, are selected to cross, Algorithm 3 describes the creation of resulting individuals H1 and H2 that we implemented for our interleaving problem.

### 3.6. Mutation

Unlike crossover, the mutation process operates in particular individuals separately. The mutation operator is used to generate new chromosomes (individuals) by (generally, in a random way) changing little portions of a given individual. In this work, we adopt the mutation operator known as Inversion Mutation (IVM), also referred to as inverted displacement mutation, as has been reported in various studies as having advantages for solving TSP [24,25]. IVM consists of taking a segment of the chromosome, inverting its values and re-introducing them in a new location.

The operator has an associated mutation probability Pm, which determines whether an individual generated after crossover mutates or not. In our proposal, we adopt Pm as a fixed input parameter of the algorithm.

In genetic algorithms, mutation’s role is to allow restoring the lost or unexplored genetic material or in the population, aiming to prevent the premature convergence to sub-optimal solutions. Indeed, such changes are necessary because, as the less adapted members of the population are discarded, some aspects of their genetic material could be definitively lost. Through random changes in chromosomes, the new individuals can explore a place of the space domain, which is not possible to reach with simple crossover.

**Algorithm 3** Order Crossover (OX1) algorithm for the interleaving problem.**Require:** Parent P1={P1(0),P1(1),⋯,P1(N.M−1)} {The first individual to cross, with P1(i)∈{1,2,⋯,N.M} and ∀i≠j, P1(i)≠P1(j).}**Require:** Parent P2={P2(0),P2(1),⋯,P2(N.M−1)} {The second individual to cross, with P2(i)∈{1,2,⋯,N.M} and ∀i≠j, P2(i)≠P2(j).}**Require:** Cutting points c1 and c2, with c1<c2, and c1≥0 and c2≤(N.M−1). 1:Initialize the first resulting individual, H1, as a vector of N.M elements. {Let us start with generating H1.} 2:Copy elements from P1(c1) to P1(c2) into H1(c1) to H1(c2). 3:i←(c2+1)mod(N.M) 4:j←i 5:**while** i≠((c1−1)mod(N.M)) **do** 6: **if** P2(j) is not in H1 **then** 7:  H1(i)←P2(j) 8:  i←(i+1)mod(N.M) 9: **end if**10: j←(j+1)mod(N.M)11:**end while**12:Initialize the second resulting individual, H2, as a vector of N.M elements. {We continue by generating H1.}13:Copy elements from P2(c1) to P2(c2) into H2(c1) to H2(c2).14:i←(c2+1)mod(N.M)15:j←i16:**while** i≠((c1−1)mod(N.M)) **do**17: **if** P1(j) is not in H2 **then**18:  H2(i)←P1(j)19:  i←(i+1)mod(N.M)20: **end if**21: j←(j+1)mod(N.M)22:**end while**

### 3.7. Replacement (Passing to the Next Generation)

Some decisions must be made about making genetic material pass to the next generation. The new population can be entirely conformed of new individuals, resulting of the genetic operators crossover and mutation, or it can be a fraction of surviving individuals from the current population (PS: Percentage of Survival). This percentage is also known as gap generation [28]. In our proposal, we adopt a PS as a fixed input parameter of the algorithm.

### 3.8. Stopping Criterion

The stopping criterion consists of defining which condition(s) make a genetic algorithm stop iterating. Usually, it consists of reaching a certain number of iterations, which is called the maximum Number of Generations (NG), and/or finding an individual with a fitness value satisfying certain characteristics. In our proposal, as the stopping criterion, we adopt the following conditions: (1) defining a maximum number of generations, NG, as a fixed input parameter of the algorithm; and (2) finding an individual with a fitness value equal to zero, which is a desired (not always possible) optimum.

### 3.9. The Genetic Algorithm

The explanations above underlie a probabilistic process that can be very complex, with multiple possible solutions. Because of the complexity of the problem, in this work, we adopt the most simple version of a GA described in Algorithm 4.

**Algorithm 4** Adopted genetic algorithm (general description). 1:t←0 2:Initialize P(0) {as defined in Section 3.2.} 3:Evaluate P(0) {with RCF described in Section 3.3} 4:**while** stopping condition {as defined in Section 3.8} is not reached **do** 5: t←t+1 6: Select P(t) from P(t−1) {with Selection strategy defined in Section 3.4} 7: Apply Crossover and Mutation over P(t) to create new individuals $P^{\prime }\left(t\right)$ {following the procedures described in Section 3.5 and Section 3.6} 8: Evaluate $P^{\prime }\left(t\right)$ {with RCF} 9: Replace P(t) from $P^{\prime }\left(t\right)$ and P(t−1) {applying description in Section 3.7}10:**end while**

## 4. Experimental Results

### 4.1. Evaluation by RCF Optimization

In this section, we analyze the enhancement in robustness allowed by our proposal, according to the RCF. Comparisons with torus automorphism in [11] and Rombaut’s SA [14] are also given. Different strategies where implemented in the Java language (Java 8 Update 91) and executed in an HP Envy 4-1152la computer with an Intel Core i5-3317U 64-bit processor, 8 GB RAM, 128GB SSD and Windows 8.1 PRO as the operating system.

For evaluation purposes, we select a number of different scenarios that represent various possible situations reported in the application field of Wireless Visual Sensor Networks (WVSN) [29,30]. We must note that, even if we restrict the number of scenarios to some particular cases due to the problem’s complexity, the number of scenarios simulated in this paper exceeds most of the related works found in the literature. Eight application scenarios were selected, differing in mask sizes (*f* matrix) and the number of elements per packet *P*. For simplicity, we consider a fixed Packet Loss Rate (PLR) of 0.2, based on previous applications, which show that this represents a usual situation in real-world WVSN, such as the work in [8] (note that this simplifies $Prob(N_{lost}=i)$ calculation in Equations (A6)–(A8) to one if *i* corresponds to the 20% of the required amount of packets and zero in any other case). In any case, we must argue that, from experimentation, we observe no significant changes in resulting RCF when varying (even greatly) PLR, for the selected evaluation scenarios.

Note that, as the optimization problem considers mask size and elements per packet as input parameters, not making any specifications about the blocks content, one combination of parameters (and the resulting output) can be applied to many application cases. For example, a mask size of 8×8 blocks with a number of elements (blocks) per packet P=6 may, represent, in the same way, the following application cases:

1. The input image has a 8×8 pixel resolution, codified in 8 bpp and divided in blocks of 1×1 pixels (by pixels) to be packetized into packets of six bytes of payload (so, six elements per packet),
2. the input image has a 8×8 pixel resolution, codified in 4 bpp (so it is compressed) and divided in blocks of 1×1 pixels (by pixels) to be packetized into packets of three bytes of payload (so, six elements per packet),
3. The image is 64×64 pixels resolution, codified in 1 bpp, divided in blocks of 8×8 and packetized into packets of 48 bytes of payload (so, six elements per packet).

The considered scenarios for the evaluation of the packetization mask generation are summarized in Table 1. As usual, each case considers a squared number of blocks, defining masks of N×N elements.

As said, selected parameters are based on related works with application in WVSN. Parameters scenarios were selected considering:

1. Maximum block size according to the JPEG standard (8×8)
2. Image sizes according to real-world WVSN resolutions 16×16, 32×32, 64×64 and 128×128.
3. Payload packets according to various reported applications, particularly: 27 bytes (as Cyclops camera firmware [31]) and 100 bytes as works in [32,33] related to IEEE 802.15.4 XBee modules [34].

For evaluation purposes, the following parameters were selected for each considered method:

- For the proposed Genetic Algorithm (GA):
  - Number of executions (number of times the algorithm was executed with the same parameters, but different random seeds): 20
  - NG=500
  - $T_{p}=150$
  - $P_{c}=0.8$
  - $P_{m}=0.03$
  - PS=0.2
- For Rombaut Simulated Annealing (RA):
  - Number of executions: 20
  - Equilibrium criteria for SA: 500 iterations
  - Equilibrium criteria for the Metropolis algorithm: 150 iterations
  - $T_{0}=1,500,000$
  - SA acceptance probability α=0.9 (according to [35,36])
- For Torus Automorphisms (TA):
  - Best parameters *k* and *n* (Equation (1)) obtained by exhaustive search for each different scenario (according with methodology in [11]). Identified parameters are presented in Table 2.

Obtained results are in terms of the average Best-obtained RCF (BRCF) from all of the iterations, for the case of GA and RA, and of the Obtained RCF (ORCF) for the case of deterministic cases Without Interleaving (WI; i.e., sequential communication line-by-line) and TA with its best parameters. Each BRCF value is obtained by averaging the best result of each of the 20 executions for the corresponding iteration number (so it represents the average best results of the genetic algorithm at a certain number of iterations, for a given number of executions).

**Figure 4:**
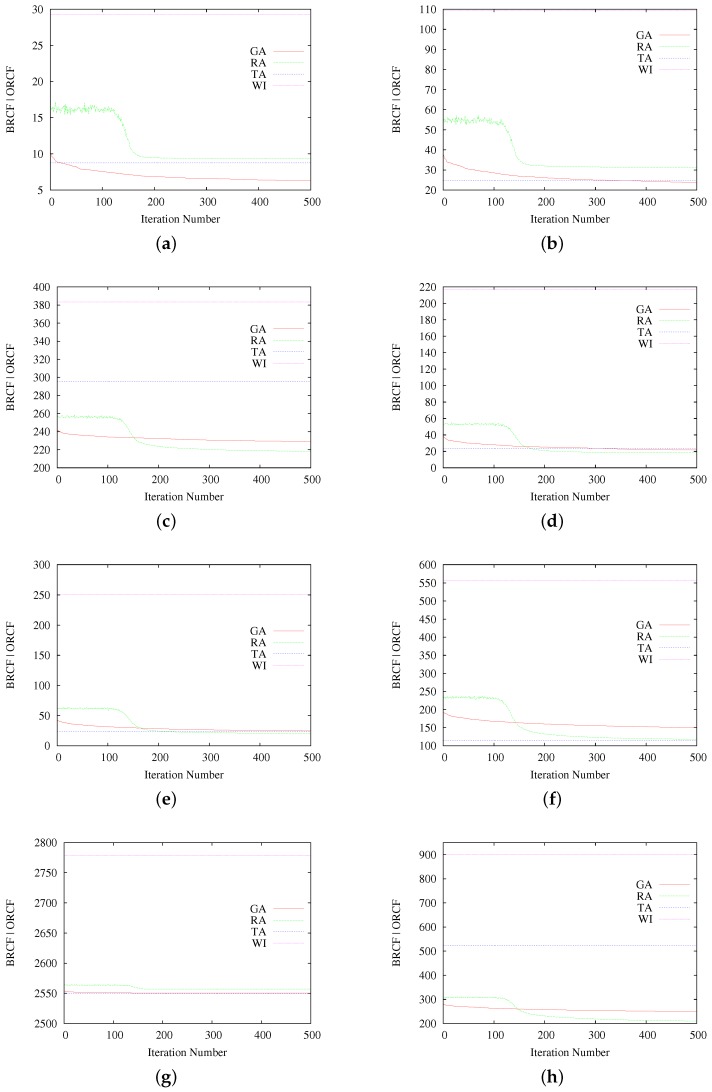
Average results for GA, SA, TA and WI for each considered scenario. (**a**) Scenario 1; (**b**) Scenario 2; (**c**); Scenario 3; (**d**) Scenario 4; (**e**) Scenario 5; (**f**) Scenario 6; (**g**) Scenario 7; (**h**) Scenario 8.

Results for each scenario are shown in Figure 4.

Obtained BRCF values for GA and RA at the considered 500 iterations, for each evaluation scenario, are shown in Table 3. ORCF for the TA and WI cases are also given. Finally, the table also shows the Average RCF (ARCF) for GA and RA, which represents the average of all of the results at the 500th iteration, for each evaluation scenario.

As a general rule, the results summarized in Table 3 (and each of the graphics in Figure 4) show better RCF results of interleaving-based methods in comparison with the non-interleaving case (WI). In terms of the best obtained RCF values, Scenarios 1, 2 and 7 show that the proposed GA presents equivalent or superior results to RA and TA, while only in Scenarios 1 and 2, the average RCF obtained by GA outperforms the other methods. In Scenarios 3, 4, 5 and 8, the best results were reported by RA. Finally, in Scenarios 6 and 7, the obtained results are slightly in favor of TA.

Regarding the performance of the proposed metaheuristic with respect to the deterministic method TA, most of the considered scenarios show the advantage of our GA, but this advantage seems to decrease as the complexity of the problem increases. Indeed, Scenarios 1 to 4 (also 8) present better performance of GA, but, when the amount of possible combinations increases (because of the mask size), TA seems to perform better. The same observation can be done for RA. In this sense, there is still an open question about the number of iterations necessary for botGA and SA, in order to reach solutions as best as possible, or a more suitable stopping criterion. What is clear (and is not said in other works) is that TA, even if effective, is far from ensuring optimal values as observed in Scenarios 3 and 8.

In the matter of the metaheuristic approaches’ comparison, even if the number of generated solutions and iterations was the same for both GA and RA, the RCF values reached for those methods differ. Indeed, only in Scenarios 1, 2 and 7, GA results overtake RA, while in Scenarios 3 to 6 and 8, RA shows the best RCF results. Regarding their behavior, GA does not present abrupt changes over the iterations, while RA presents a temporary big acceleration on the improvement of the calculated RCF around Iteration 150.

From these results, even if GA achieved good RCF values in various cases, better or equal to other methods, in most of the scenarios, the better results were achieved by RA or TA. Even though, it is difficult to evaluate this disadvantage because there are no recipes to assess a difference of one RCF point. Indeed, results are in terms of an absolute value coming from penalty functions, but it results in being hard to estimate which difference is significant. We expect to find a valuable answer by using the generated packetization masks for communicating images through unreliable channels, as commented in the following subsection. Anyway, the first results are encouraging, and future works could enhance these indicators by applying other kinds of operators or (event) computing approaches, such as parallel or distributed computing.

**Figure 5:**
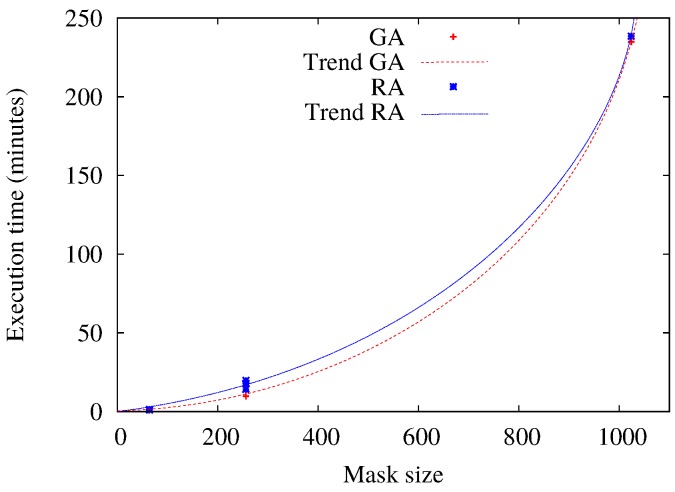
Comparison of the execution times between RA and GA. Pointed lines are fitted curves to the obtained samples.

About the execution times, the eventual enhancing of the resulting patterns allowed by using metaheuristics contrasts with their required time of execution. Indeed, the calculation of TA (and other deterministic methods) is performed in a few milliseconds [11]. Actually, TA complexity is low enough to be implemented in very resource-constrained hardware [8]. Simulation times for RA and GA are summarized in Figure 5. Of course, execution times depend on the hardware adopted and the implementation itself. For the executions of the selected scenarios, our implementations of RA and GA spend almost the same time. For example, for a 32×32 mask (mask size = 1024), the obtained average execution times are around 250 and 268 minutes for GA and RA, respectively. This is much too complex for being applied in real time or resource-constrained devices, even if enhancements can be allowed by using other kinds of platforms (like parallel or distributed computing), but the real goal of applying metaheuristics for this kind of problem is to provide optimal parameters for off-line analysis and design. In particular, this kind of solution can be valuable for, e.g., providing comparison references to evaluate how “optimal”a packetization mask, obtained by deterministic/fast methods, is, the off-line design of the optimal packetization mask for sensor hardware or interleaver chip designs, etc.

### 4.2. Image Quality Assessment Results

Results reported in Section 4.1 allow one to observe the behavior of our GA compared with the considered related works RA and TA, in terms of their reached RCF values. From all of the executions, from each of the considered method, we select the best obtained patterns (masks) in order to evaluate the improvement in the quality of images transmitted through unreliable media using simulation framework Sim-LIT 2.0 [37]. Sim-LIT allows analyzing the effects of packet loss in communicated images, providing results in terms of the well-known quality assessment metric PSNR (Peak Signal-to-Noise Ratio). As said above, the considered scenarios are based on WVSN, so we select images in typically adopted resolutions and color depths. For testing purposes, we select:

- eight different loss rates: 10%, 20%, 30%, 40%, 50%, 60%, 70%, and 80%,
- 200 different, randomly generated, loss distributions, for each different PLR, and
- eight different uncompressed (raw) 8 bpp grayscale images.

**Figure 6:**
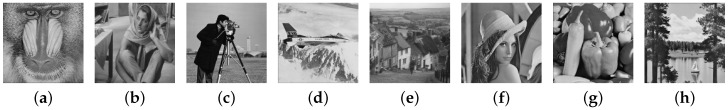
Images adopted for image quality assessment: (**a**) Baboon, (**b**) Barbara, (**c**) Cameraman, (**d**) F-16, (**e**) Goldhill, (**f**) Lena, (**g**) Peppers and (**h**) Sailboat.

For testing, and in order to consider different kinds of images, we select classical testing images Baboon, Barbara, Cameraman, F-16, Goldhill, Lena, Peppers and Sailboat, which are depicted in Figure 6.

For estimating the intensities of missing pixels, we adopt the average value of an eight-connected pixel neighborhood, as the error concealment technique.

Finally, the considered parameters defining the simulation scenarios are summarized in Table 4.

Simulation results are presented separately by packetization mask and image. Without loss of generality and due to the enormous quantity of obtained data, we describe only results analysis Scenario A and examples of reconstructed images for Scenario B. We must argue that the rest of the scenarios and executed simulations behave in a similar way. With this, we attempt to provide results to validate the suitability of the selected metric.

**Figure 7:**
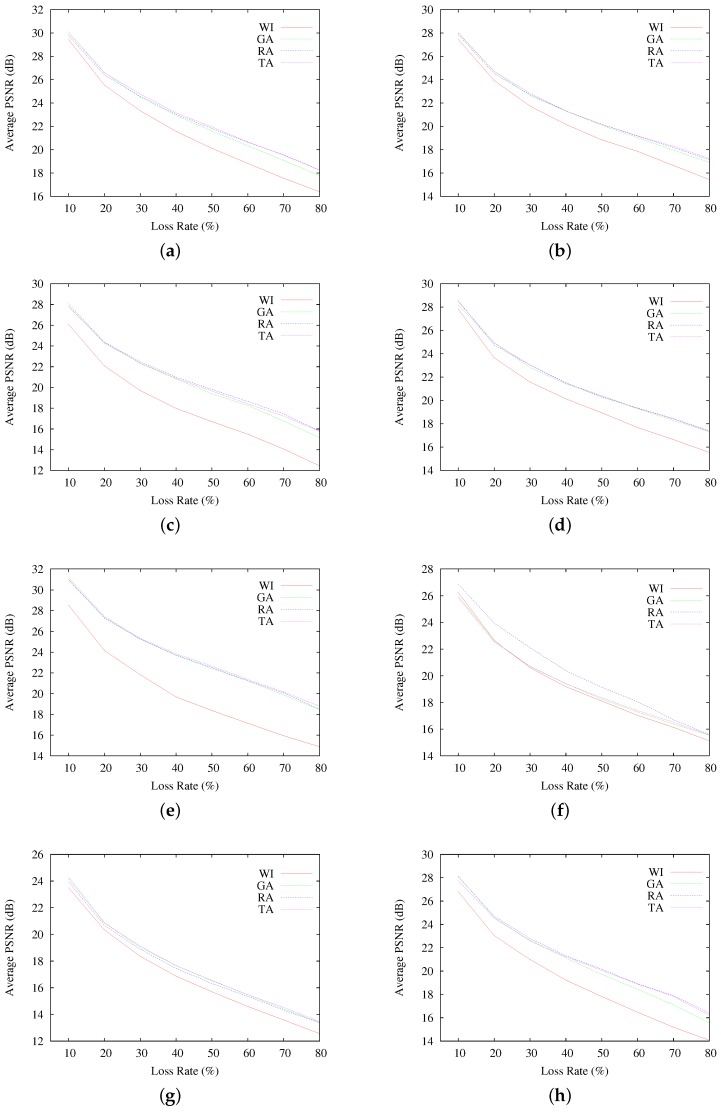
Average PSNR results for Scenario A, per testing image. (**a**) results for Baboon; (**b**) results for Barbara; (**c**) results for Cameraman; (**d**) results for F-16; (**e**) results for Goldhill; (**f**) results for Lena; (**g**) results for Peppers; (**h**) results for Sailboat.

**Figure 8:**
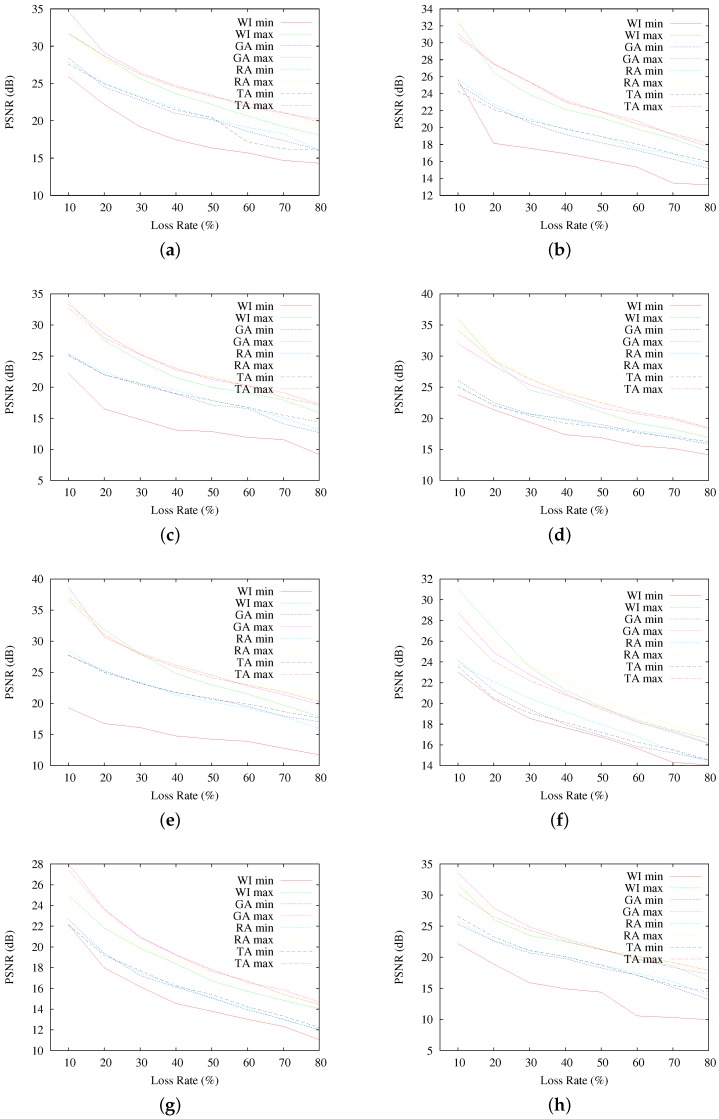
Highest and lowest PSNR results for Scenario A, per testing image. (**a**) results for Baboon; (**b**) results for Barbara; (**c**) results for Cameraman; (**d**) results for F-16; (**e**) results for Goldhill; (**f**) results for Lena; (**g**) results for Peppers; (**h**) results for Sailboat.

Figure 7 presents the average PSNR of reconstructed images, as a function of the loss rate. Each graphed average result corresponds to the 200 resulting images at each different PLR. As seen, in terms of average PSNR, interleaving-based communication using GA, RA and TA indeed achieves better qualities of the reconstructed images than the sequential communication case (WI). However, the obtained quality results do not allow one to observe discernible differences among the selected interleaving schemes, resulting in average PSNR fairly similar for all GA, RA and TA, even if they obtain quite different RCF results. A similar observation can be made by looking at the results in Figure 8, where the highest and lowest resulting PSNR for all of the reconstructed images are presented.

Reconstructed images from Scenario B in Table 5 show the effect of data loss, when applying a packet loss rate of 50%, per each of the adopted images used in the simulations. These results correspond to the highest reached PSNR from the 200 loss distributions (at a 50% PLR) after applying the best GA packetization mask and WI communication. As seen, even if the resulting RCF for the WI and GA cases are distant, the obtained image qualities are similar, as when high PLR occurs, a good packetization in terms of RCF does not ensure good reconstruction of missing information. This is rather a combination of factors, including error concealment and the losses’ nature.

Table 6 shows the Baboon image reconstructed after applying different loss rates (10%, 30%, 50% and 70%), for the WI case and the interleaving-based schemes based on GA, RA and TA according to their best resulting packetization masks (from the 200 distributions), corresponding to Scenario B. As the previous case, even if the resulting RCF are different, PSNR results are fairly similar.

**Figure 9:**
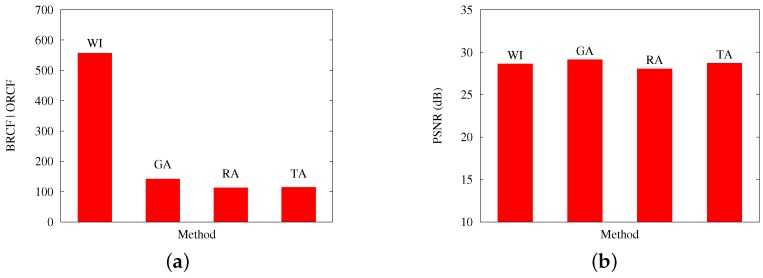
Results from Scenario A, at a 20% packet loss. The figure contrasts the (**a**) best RCF values reached with packetization masks generated by GA and the other applied methods; and (**b**) the PSNR values reached on the evaluation of image quality for the image Baboon.

**Figure 10:**
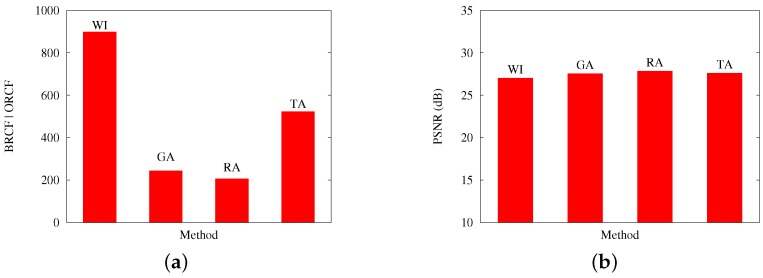
Results from Scenario B, at a 20% packet loss. The figure contrasts (**a**) the best RCF values reached with packetization masks generated by GA and the other applied methods; and (**b**) the PSNR values reached on the evaluation of image quality for the image Baboon.

The previous comments are reinforced by Figure 9 and Figure 10, which contrast, for Scenarios A and B, respectively: (a) the best obtained RCF per algorithm; and (b) the best PSNR results. Both cases present results with an applied loss rate of 20%

## 5. Conclusions

The problem of finding a packetization pattern with genetic algorithms was tackled. This technique allows one to engage the huge amount of possible solutions to the problem, when robustness against packet loss is required. This problem has been treated very little in the literature, but it has enormous application in image communication systems where resource savings are required.

Patterns generated by the proposed GA were evaluated using as the fitness function the cost function defined by Rombaut et al. in [14], which evaluated a resulting packetization mask in accordance with three main properties. The proposed GA, along with the proposed SA by Rombaut et al. and a TA-based interleaver, was tested in different scenarios using parameters from WVSN works. Results in terms of obtained RCF (the selected fitness metric) present significant favor for interleaving methods against simple sequential non-interleaved packetization. Research results show that, however, no determinant conclusion can be made about what implemented interleaving method is best suited, as they present similar results, some in favor of GA and some in favor of the other. Moreover, there is no basis of comparison to determine how much points of RCF represent a significant difference when comparing two different masks. In terms of image quality assessment, research results allow one to show that, even if clear differences can be obtained by RCF, visual observation and PSNR results do not vary much in most of the considered cases. Most of these conclusions are due to the fact that, for the inability to access better computing equipment, we limited our study to cases where the size of the data blocks is high in comparison to the matrix sizes, so the amount of possible combinations is low. In any case, our results put in evidence the need for evaluation metrics that consider these facts, providing useful information about the suitability of packetization patterns as a technique for enhancing robustness in image communication. In this sense, it is expected to prove the statistical representativity of the results, which is no treated in the literature. To be more useful, a proper evaluation function should represent results in terms of image quality, but also, the comparison methodologies must be validated. Other image quality metrics will be considered in future works, such as the structural similarity index (SSIM). Furthermore, new techniques for finding optimal patterns are required; as future work, we expect to study other kinds of a approaches, such as Particle Swarm Optimization (PSO) and the Greedy Randomized Adaptive Search Procedure (GRASP). The proposed GA, even if it does not overcome the other interleaving functions, presents promising results at the level of other related works. Further works will consider the application of parallel computing and other genetic operators. As a final comment, most of these issues are still open, presenting challenging problems to the research community.

## Figures and Tables

**Table 1:** Evaluation scenarios for evaluating packetization mask generation.

Scenario	Mask Size (N×N)	Elements per Packet (*P*)
1	8 × 8	6
2	8 × 8	25
3	16 × 16	6
4	16 × 16	25
5	16 × 16	27
6	16 × 16	100
7	32 × 32	6
8	32 × 32	25

**Table 2:** Best parameters found for TA for each evaluation scenario.

Scenario	*k*	*n*
1	4	1
2	3	1
3	1	7
4	4	2
5	12	6
6	1	3
7	1	2
8	12	9

**Table 3:** RCF results for the test scenario.

Scenario	WI ORCF	GA BRCF	GA ARCF	RA BRCF	RA ARCF	TA ORCF
1	29.2727	5.8364	6.3186	9.3091	9.3363	8.7455
2	109.3333	20.0000	23.7894	31.3333	31.3333	24.6667
3	383.2625	227.4618	229.1624	217.495	218.1787	295.495
4	217.3091	20.0364	21.6105	18.4364	18.5327	23.3455
5	250.8222	22.7778	24.6070	20.1778	20.5511	23.7778
6	557.3333	141.3333	150.0000	112.6667	117.7333	114.0000
7	2778.2573	2550.3018	2550.3139	2557.0901	2557.0901	2550.3018
8	898.7805	244.3707	249.9034	206.7951	209.8641	522.9854

**Table 4:** Scenarios corresponding to the evaluation based on other parameters.

Scenario	Image Resolution (pixels)	Block Size (bytes)	Payload (bytes)	Partitions (Elements)	Number of Packets to Transmit	Mask Size
A	16×16	1×1	27	27	10	16×16
B	64×64	2×2	100	25	41	32×32

**Table 5:** Obtained images with the highest reached PSNR after simulations with 50% PLR, Scenario B.

Original	WI Case (RCF = 898.7805)		GA-Based Case (RCF = 244.3707)	
Image	Image with Losses	Reconstructed Image	Image with Losses	Reconstructed Image
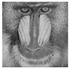	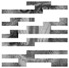	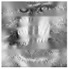	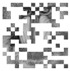	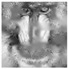
Baboon		PSNR: 21.6493 dB		PSNR: 22.2398 dB
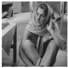	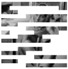	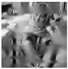	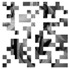	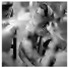
Barbara		PSNR: 20.7704 dB		PSNR: 20.9886 dB
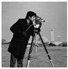	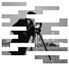	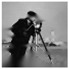	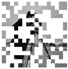	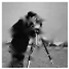
Cameraman		PSNR: 22.1295 dB		PSNR: 21.8912 dB
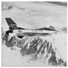	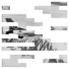	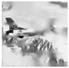	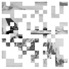	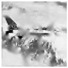
F-16		PSNR: 20.4662 dB		PSNR: 20.5709 dB
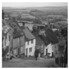	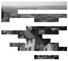	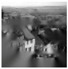	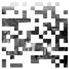	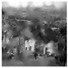
Goldhill		PSNR: 22.4507 dB		PSNR: 23.2100 dB
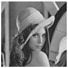	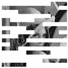	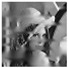	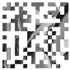	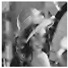
Lena		PSNR: 20.9367 dB		PSNR: 20.5371 dB
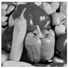	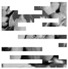	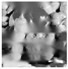	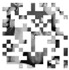	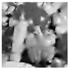
Peppers		PSNR: 18.1289 dB		PSNR: 18.9816 dB
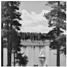	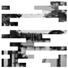	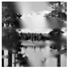	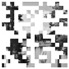	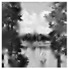
Sailboat		PSNR: 18.7318 dB		PSNR: 19.4676 dB

**Table 6:** Reconstructed images with best PSNR reached after simulations applying masks from the WI, GA, RA and TA cases, Scenario B.

Loss Rate	Reconstructed Images			
	WI Case	GA Case	RA Case	TA Case
	(RCF = 898.7805)	(RCF = 244.3707)	(RCF = 206.7951)	(RCF = 522.9854)
10%	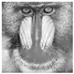	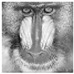	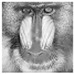	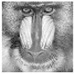
	PSNR: 30.7916 dB	PSNR: 31.1916 dB	PSNR: 30.9109 dB	PSNR: 31.0231 dB
30%	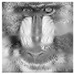	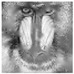	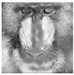	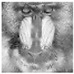
	PSNR: 25.2869 dB	PSNR: 25.6539 dB	PSNR: 25.4085 dB	PSNR: 25.7146 dB
50%	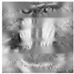	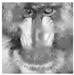	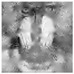	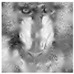
	PSNR: 21.6493 dB	PSNR: 22.2398 dB	PSNR: 22.1582 dB	PSNR: 22.5291 dB
70%	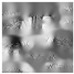	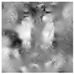	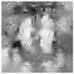	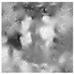
	PSNR: 19.2372 dB	PSNR: 19.6966 dB	PSNR: 19.6124 dB	PSNR: 19.8461 dB

## Abbreviations

The following abbreviations are used in this manuscript:

ARCF	Average RCF (from a set of values)
BRCF	Best-obtained RCF (from a set of values)
GA	Genetic Algorithm
IVM	Inversion Mutation
ORCF	Obtained RCF
PLR	Packet Loss Rate
PSNR	Peak Signal to Noise Ratio
RA	Rombaut et al.’s Algorithm
RCF	Rombaut’s Cost Function
SA	Simulated Annealing
TA	Torus Automorphisms
TSP	Traveling Salesman Problem
WI	Without Interleaving
WVSN	Wireless Vision Sensor Networks

## Appendix A. Calculation of Terms of RCF

According to [14], RCF terms are calculated as follows:

(A1) $$\begin{array}{ccc} Q_{1}\left(B\right) & = & \frac{1}{P}\sum_{i=0}^{P-1}\sum_{k=0}^{\#S_{i}^{B}-1}\sum_{\begin{array}{c} l=0\\ l\neq k \end{array}}^{\#S_{i}^{B}-1}C\left(d\left(s_{i,k}^{B},s_{i,l}^{B}\right)\right) \end{array}$$

(A2) $$\begin{array}{cc} Q_{2}\left(B\right)= & \frac{1}{P(P-1)}\sum_{i=0}^{P-1}\sum_{\begin{array}{c} j=0\\ i\neq j \end{array}}^{P-1}\sum_{k=0}^{\#S_{i}^{B}-1}\left[\sum_{l=0}^{\#S_{j}^{B}-1}C\left(d\left(s_{i,k}^{B},s_{j,l}^{B}\right)\right)\cdot 1\left(\sum_{l=0}^{\#S_{j}^{B}-1}1\left(d\left(s_{i,k}^{B},s_{j,l}^{B}\right)\leq d_{N}\right)\geq 2\right)\right] \end{array}$$

(A3) $$\begin{array}{ccc} Q_{3}\left(B\right) & = & \frac{1}{P(P-1)}\sum_{i=0}^{P-1}\sum_{\begin{array}{c} j=0\\ i\neq j \end{array}}^{P-1}\sum_{n=1}^{N}\left(C\left(d_{n}\right)\left|\sum_{k=0}^{\#S_{i}^{B}-1}\sum_{l=0}^{\#S_{j}^{B}-1}1\left(d\left(s_{i,k}^{B},s_{j,l}^{B}\right)=d_{n}\right)-N_{n,j}\right|\right) \end{array}$$

where:

#Si: is the number of elements in packet $S_{i}$.
si,kB: is the position of the *k*-th element in the packet *i* of the partition.
d(s,t): is the distance between *s* and *t* given by:
  (A4) $$d\left(s,t\right)=\sqrt{\left(x_{s}-x_{t}\right)\;\mathrm{mod}\;M+\left(y_{s}-y_{t}\right)\;\mathrm{mod}\;N}$$
C(d): is the cost associated with the distance *d*. This cost function C(d) is directly related to the error concealment technique (the technique that allows estimating the intensities of a lost block by using the correctly received ones). In this work, we adopt as the error concealment technique the average of the eight directly connected (and, of course, correctly received) blocks, so we define C(d) as:
  (A5) $$C\left(d\right)=\left\{\begin{array}{cc} 1 & \mathrm{if}\;d=1\vee d=\sqrt{2}\\ 0 & \mathrm{otherwise} \end{array}\right.$$
1(expr): evaluates expression expr. If expr is true, it returns 1 (one); otherwise, it returns zero (zero).
dN: according to the error concealment technique, is the distance by which the more distant element needed to reconstruct a missing block is from that block in the original image. In this case, it is considered N=3, $d_{N}=2$.
Nn,j: is the amount of elements that a packet should have.

Factors α, β and γ are calculated as:

(A6) $$\begin{array}{ccc} \alpha & = & \sum_{u=0}^{P}iProb\left(N_{lost}=i\right) \end{array}$$

(A7) $$\begin{array}{ccc} \beta & = & \sum_{i=0}^{P}i\left(i-1\right)Prob\left(N_{lost}=i\right) \end{array}$$

(A8) $$\begin{array}{ccc} \gamma & = & \sum_{i=0}^{P}i\left(i-1\right)Prob\left(N_{lost}=i\right) \end{array}$$

where $Prob(N_{lost}=i)$ is the probability of, according to the considered packet loss model, losing *i* packets.
